# Determinants of Epstein-Barr virus-positive gastric cancer: an international pooled analysis

**DOI:** 10.1038/bjc.2011.215

**Published:** 2011-06-07

**Authors:** M C Camargo, G Murphy, C Koriyama, R M Pfeiffer, W H Kim, R Herrera-Goepfert, A H Corvalan, E Carrascal, A Abdirad, M Anwar, Z Hao, J Kattoor, E Yoshiwara-Wakabayashi, Y Eizuru, C S Rabkin, S Akiba

**Affiliations:** 1 Division of Cancer Epidemiology and Genetics, National Cancer Institute, National Institutes of Health, 6120 Executive Boulevard., Rockville, MD 20852, USA; 2 Division of Epidemiology and Biostatistics, University of Illinois, 1603 W. Taylor Street, Chicago, IL 60612, USA; 3 Department of Epidemiology and Preventive Medicine, Center for Chronic Viral Diseases, Graduate School of Medical and Dental Sciences, Kagoshima University, 8-35-1 Sakuragaoka, Kagoshima 890-8544, Japan; 4 Department of Pathology, Seoul National University College of Medicine, 28 Yongon-dong, Chongno-gu, Seoul 110-799, South Korea; 5 Department of Pathology, National Cancer Institute, Av. San Fernando #22, Col. Sección XVI, Tlalpan, 14080 Mexico, D.F., Mexico; 6 Department of Hematology and Oncology, Pontificia Universidad Catolica, 85 Lira Street, Santiago 133-202, Chile; 7 Cali Cancer Registry, Universidad del Valle, Calle 4B No. 36-00 Edificio 116, Cali, Colombia; 8 The Cancer Institute, Tehran University of Medical Sciences, PO Box 14155-6447, 1417613151 Tehran, Iran; 9 Department of Pathology, The Second Affiliated Hospital, Guangzhou Medical College, Haizhu District, Guagngzhou 510260, Guangdong, China; 10 Department of Pathology, Regional Cancer Center, Medical College Campus, Trivandrum 695011, Kerala, India; 11 Policlinico Peruano Japones, Av. Gregorio Escobedo 783 Jesus Maria, Lima, Peru; 12 Division of Oncogenic and Persistent Viruses, Center for Chronic Viral Diseases, Graduate School of Medical and Dental Sciences, Kagoshima University, 8-35-1 Sakuragaoka, Kagoshima 890-8544, Japan

**Keywords:** gastric cancer, EBV, prevalence, pooled analysis

## Abstract

**Background:**

Meta-analyses of the published literature indicate that about 9% of gastric cancers contain Epstein-Barr virus (EBV), with consistent and significant differences by sex and anatomic subsite. This study aimed to identify additional determinants of EBV positivity and their joint effects.

**Methods:**

From 15 international populations with consistent laboratory testing for EBV, we pooled individual-level data for 5081 gastric cancer cases including information on age, sex, subsite, histologic type, diagnostic stage, geographic region, and period of diagnosis. First, we combined population-specific EBV prevalence estimates using random effects meta-analysis. We then aggregated individual-level data to estimate odds ratios of EBV positivity in relation to all variables, accounting for within-population clustering.

**Results:**

In unadjusted analyses, EBV positivity was significantly higher in males, young subjects, non-antral subsites, diffuse-type histology, and in studies from the Americas. Multivariable analyses confirmed significant associations with histology and region. Sex interacted with age (*P*=0.003) and subsite (*P*=0.002) such that male predominance decreased with age for both subsites. The positivity of EBV was not significantly associated with either stage or time period.

**Conclusion:**

Aggregating individual-level data provides additional information over meta-analyses. Distinguishing histologic and geographic features as well as interactions among age, sex, and subsite further support classification of EBV-associated gastric cancer as a distinct aetiologic entity.

Gastric cancer is the second leading cause of cancer-related deaths worldwide (Parkin *et al*, 2005). Chronic *Helicobacter pylori* infection is considered to be the initiating factor in gastric carcinogenesis (International Agency for Research on Cancer, 1994), although human genetic polymorphisms and environmental factors also modulate risk of neoplasia (Correa *et al*, 2006; Hamajima *et al*, 2006).

Epstein-Barr virus (EBV) is a ubiquitous infectious agent which has been causally linked to the development of several malignancies, including Burkitt's lymphoma, immunosuppression-related lymphoma, Hodgkin's lymphoma, and nasopharyngeal carcinoma (International Agency for Research on Cancer, 1997). Although some cases of gastric adenocarcinoma harbour EBV infection (Takada, 2000; Akiba *et al*, 2008), it remains unclear whether the presence of EBV is a cause or a consequence of neoplastic changes.

To expand on our recent meta-analysis of EBV prevalence in gastric cancer (Murphy *et al*, 2009), we have conducted a pooled analysis of individual-level data on variables for which the published aggregate data were insufficient for meta-analysis. In addition, we aimed to investigate whether EBV positivity is modified by the joint effect of these determinants.

## Materials and methods

### Data sources

Over the past 10 years, one of us (SA) has participated in multiple international collaborations assessing the presence of EBV in gastric cancer tumours in Asia and Latin America. In general, these studies followed a common protocol evaluating EBV infection by EBV-encoded small RNA *in situ* hybridisation. In the present reanalysis, we have combined individual-level data of 12 of these studies from areas with differing risk profiles for gastric cancer (Corvalan *et al*, 2001; Koriyama *et al*, 2001, 2004; Hao *et al*, 2002; Kattoor *et al*, 2002; Carrascal *et al*, 2003; Lee *et al*, 2004; Anwar *et al*, 2005; Herrera-Goepfert *et al*, 2005; Yoshiwara *et al*, 2005; Campos *et al*, 2006; Abdirad *et al*, 2007). Since none of these studies was conducted in North America or Europe, these geographic areas were not represented in our analysis. We included information on EBV status, sex, age at diagnosis, geographic region, histologic type, anatomic subsite, diagnostic stage, and year of diagnosis. Two of the identified studies (Koriyama *et al*, 2001; Hao *et al*, 2002) reported findings from more than one population group, and thus, data on 15 distinct populations were analysed.

### Statistical analyses

Given the limited sample size in some studies, EBV prevalence and corresponding standard errors (s.e.) were calculated by the Wilson method (Wilson, 1927) for each population, overall and separately for the following five covariables: age at diagnosis (treated as a continuous and as a categorical variable), sex (male *vs* female), anatomic subsite (antrum *vs* other subsites; overlapping cases were excluded), diagnostic stage (early *vs* advanced), and Lauren histologic type (diffuse *vs* intestinal). The diffuse subtype included the following Japanese histologic classifications: solid type poorly differentiated adenocarcinoma (por1), non-solid type poorly differentiated adenocarcinoma (por2), signet-ring cell carcinoma (sig), and lymphepithelial-like carcinoma (LE). The intestinal subtype included the following: well-differentiated type tubular adenocarcinoma (tub1), moderately differentiated type tubular adenocarcinoma (tub2), papillary adenocarcinoma (pap), and mucinous adenocarcinoma (muc). Population-specific log odds ratios (ORs) and 95% confidence intervals (CIs) of EBV positivity were calculated for the same five covariables using standard logistic regression models. Strata with no EBV-positive cases were assigned an arbitrary prevalence of 1% to allow calculation of ORs and for the subsequent meta-analysis.

Random effects meta-analysis (DerSimonian and Laird, 1986) was used to pool overall log prevalence estimates, with s.e. (log prevalence) calculated by the Delta-method as 1/prevalence × s.e. (prevalence), and to pool log ORs for covariables. Between study heterogeneity was assessed using the *I*^2^ and *Q* statistics, with *I*^2^ >50% or *P*_Q_<0.05 indicating heterogeneity (Deeks, 2002; Higgins and Thompson, 2002).

To increase statistical precision, pooled ORs were also estimated for aggregated data using logistic regression models that included a random population-specific intercept. This approach avoided the computational requirement for non-zero prevalence for all subgroups. In addition to the covariables-listed above, we also assessed the separate effects of geographic region (Americas *vs* Asia), estimated national gastric cancer incidence rates in 2008 (<10, 10–19.9, and ⩾20 cases per 100 000 population; Ferlay *et al*, 2010), year of diagnosis, and decade of birth. Year of diagnosis was treated alternatively as a continuous or categorical (⩽1959, 1960–1969, 1970–1979, 1980–1989, and ⩾1990) variable. Decade of birth was also treated as either continuous or categorical (⩽1919, 1920–1929, 1930–1939, 1940–1949, 1950–1959, and ⩾1960). Additionally, age, year of diagnosis, and decade of birth were tested for non-linear associations with EBV positivity by including the squared terms of the variables in the respective models. Two-sided *P*-values for associations <0.05 were considered statistically significant. Pooled ORs for the meta-analytic and aggregated approaches were generally similar, so only the aggregated estimates are presented.

To assess interactions and evaluate confounding, we performed multivariable aggregated analysis, excluding the terms for decade of birth and national incidence due to collinearity with age and period and with region, respectively. To evaluate effect modification, we generated cross-product terms for the six pairwise combinations of the four variables significantly associated with EBV positivity (i.e., age at diagnosis, sex, anatomic subsite, and histologic type). In the final models, age at diagnosis was treated as a continuous variable and adjustments were included for stage, decade of diagnosis, and geographic region. Wald *χ*^2^-tests were used to assess interaction terms, with *P*-values corrected for multiple comparisons of <0.0083 (0.05/6) considered statistically significant. Estimated parameters from the final regression model were used to calculate age-specific prevalence of EBV positivity by sex and anatomic subsite, as well as age- and subsite-specific ORs of EBV positivity for males compared with females. All statistical analyses were performed in Stata 10.0 (Stata Corp, College Station, TX, USA).

## Results

**Figure 1:**
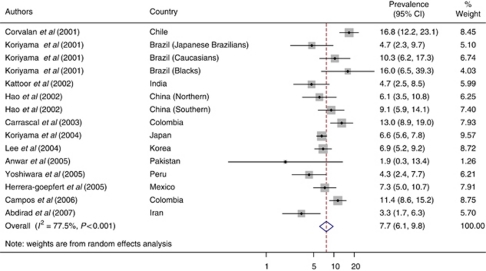
Estimated prevalence (95% CIs) of EBV positivity in gastric cancers from 15 populations.

A total of 5081 gastric cancer cases from 15 different populations contributed to these analyses (Table 1). The meta-analytic estimated prevalence of EBV positivity was 7.7% overall (95% CI: 6.1–9.8), with high heterogeneity among studies (*I*^2^=77.5%, *P*_Q_<0.001; Figure 1).

In unadjusted analyses (Table 2), the following association patterns emerged: (a) tumours in young subjects were more likely to be EBV positive than those in older subjects, (b) tumours in males were more than twice as likely to be EBV positive than tumours in females, (c) non-antral tumours were more likely to be EBV positive than those arising in the antrum, (d) diffuse-type tumours were almost twice as likely to be EBV positive than tumours of intestinal histology, and (e) EBV prevalence was similar in early and advanced tumours. Furthermore, EBV positivity did not significantly differ by decade of birth (ORs for comparison with 1910s or earlier: 1.3 for 1920s, 1.1 for 1930s, 1.2 for 1940s, 1.5 for 1950s, and 1.6 for 1960s or later) or decade of diagnosis (ORs using 1990 or later as referent: 0.7 for ⩽1959, 1.2 for 1960s, 1.0 for 1970s, and 1.3 for 1980s). However, EBV positivity was significantly higher for studies conducted in the Americas as compared with those in Asia (OR: 1.7, 95% CI: 1.1–2.7) and marginally lower in countries with gastric cancer incidence rates <10 or ⩾20 cases per 100 000 population as compared with 10–19.9 cases per 100 000 population (OR: 0.5, 95% CI: 0.3–1.0 and OR: 0.7, 95% CI: 0.4–1.1, respectively). Inclusion of squared terms for age, decade of birth and period did not significantly improve the fit of models with linear terms for these variables.

**Figure 2:**
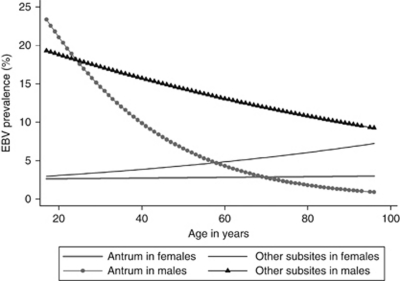
Fitted age-specific prevalence of EBV positivity in gastric tumours, by sex and anatomic subsite.

The multivariable aggregated analyses identified four pairwise interactions at the *P*<0.05 level: sex and anatomic subsite (*P*-value=0.002), age and sex (*P*-value=0.003), age and anatomic subsite (*P*-value=0.011), and age and histologic type (*P*-value=0.013), with only the first two statistically significant as corrected for multiple comparisons. Prevalence of EBV positivity decreased with age among men, more steeply for tumours localised to the antrum than for those localised to other anatomic subsites (Figure 2). In contrast, there was no significant variation by age among women, although tumours in non-antral subsites had slightly higher EBV prevalence. In terms of the risk estimates, male predominance decreased with age for both subsites (Table 3).

In a final model that included the two significant interactions, we confirmed the associations of EBV positivity with diffuse-type histology (OR: 2.0, 95% CI: 1.5–2.6) and with studies from the Americas (OR: 2.3, 95% CI: 1.5–3.7). Differences by diagnostic stage or period of diagnosis were not statistically significant.

## Discussion

We analysed individual-level participant data on 5081 subjects with gastric cancer from international studies we previously conducted with a consistent approach to EBV testing. As compared with our individual reports, the present reanalysis had greater statistical power for robust observations. The magnitude of the observed pooled prevalence (7.7% 95% CI: 6.1–9.8%) and the degree of heterogeneity among our studies were similar to that reported in previous meta-analyses of the published literature (Lee *et al*, 2009; Murphy *et al*, 2009; Li *et al*, 2010). Moreover, this pooled analysis also evaluated associations with additional variables that were not included in prior meta-analyses, provided adjusted estimates, and was able to examine interactions.

Previous reports have indicated that EBV-positive gastric tumours tend to be proximally located, and account for a greater proportion of cases in males than in females (Murphy *et al*, 2009). However, few studies have found variation by age (Chang *et al*, 2003; van Beek *et al*, 2004). With our large data set, we found that EBV positivity varies jointly by age, sex, and anatomic subsite, interactions that may be obscuring a main effect of age. The young predominance in males and the countervailing age trend in females suggest the importance of occupational, hormonal, and/or lifestyle factors in the aetiology of EBV-associated gastric cancer.

*H. pylori* infection preferentially colonises the antrum (Martín-de-Argila *et al*, 1997), so the contrasting localisation of EBV-positive gastric cancer to non-antral subsites suggests possible antagonism between the two infectious agents. An *in vitro* study directly examining this interaction found that reactive products from *H. pylori* infection trigger EBV reactivation in latently infected gastric epithelial cells (Minoura-Etoh *et al*, 2006).

Two reports encompassed in the present analysis have examined variation in EBV prevalence of gastric tumours over time or birth cohort. Abdirad *et al* (2007) noted a cluster of EBV positivity in cancers of a specific birth cohort (Iranians born 1928–1930). However, Herrera-Goepfert *et al* (2005) found similar EBV prevalence in Mexican cases diagnosed during 1980–1989 as compared with those diagnosed in 1990–2000. Our pooled analysis, using data on cases diagnosed between 1949 and 2004, did not find consistent changes over time.

An inverse relationship has previously been suggested between the background population incidence of gastric cancer and EBV prevalence in the tumours (Murphy *et al*, 2009). In our data, EBV positivity was similar for cases in countries with high and low background incidence, but significantly higher in tumours from the Americas as compared with those from Asia. While we cannot distinguish their independent effects, EBV-positive gastric cancer may differ by geographic region rather than with background incidence *per se*.

While results from previous meta-analyses disagreed regarding associations with histologic subtype (Lee *et al*, 2009; Murphy *et al*, 2009; Li *et al*, 2010), we found a higher EBV prevalence in diffuse-type tumours. Histologic classification differs among pathologists (Borchard, 1990) and our pooled analysis may have had more consistent laboratory methods and pathologic review than the varied approaches in the published literature.

Although it has been suggested that EBV-positive gastric cancer cases may have a better prognosis than EBV-negative cases, previous individual reports are limited and somewhat inconsistent. Recently, Song *et al* (2010) reported a survival advantage for certain histologic subgroups of EBV-associated gastric cancer in a large Korean series, but there was no significant survival advantage for EBV-positive tumours overall. A meta-analytic review by Lee *et al* (2009) including seven studies did not find associations with the clinicopathologic prognostic indicators clinical stage, depth of invasion, or lymph node metastasis. Our null finding for clinical stage was consistent with this meta-analysis and, importantly, our estimates were also adjusted for possible confounders.

Although the specific role of EBV in gastric carcinogenesis has not yet been elucidated, several lines of evidence support its aetiological significance. Epstein-Barr virus-positive gastric carcinomas exhibit uniform presence of monoclonal viral episomes in the tumour cells (Ott *et al*, 1994). Epstein-Barr virus-positive gastric carcinomas also display distinct clinical and genetic features relative to EBV-negative tumours (zur Hausen *et al*, 2000; van Beek *et al*, 2004). In addition, some studies have found elevated serum antibodies against viral capsid and nuclear antigens preceding development of preneoplastic and neoplastic gastric lesions (Levine *et al*, 1995; Schetter *et al*, 2008).

Histologic specificity and geographic variation suggest that EBV-positive gastric cancer is a distinct entity. The pairwise interactions that we found among age, sex, and anatomic subsite are novel and should be explored further. Our findings reveal a complex interplay of factors influencing EBV's presence in gastric tumours and may provide important clues to understanding its aetiologic significance.

## Figures and Tables

**Table 1:** Selected characteristics of the study populations

							**Tumour characteristics (%)** a		
**Country**	**Authors, year of publication**	**GC incidence rate in both sexes, ASR (W)^b^**	**Sample size**	**Study period**	**Age in years, mean (s.d.)**	**% Males**	**Antrum location**	**Diffuse type^c^**	**Early stage**
Brazil	Koriyama *et al* (2001)	10.9	126 (Caucasians)	1949–1988	54 (12)	67	89	48	8
			25 (Blacks)	1949–1988	49 (13)	72	94	42	8
			149 (Japanese Brazilians)	1949–1988	56 (12)	66	75	53	10
Chile	Corvalan *et al* (2001)	17.9	185	1993–1997	61 (13)	65	35	38	22
China	Hao *et al* (2002)	29.9	180 (Northern)	1991–2000	59 (12)	73	35	50	5
			198 (Southern)	1987–2000	58 (12)	63	53	57	4
Colombia	Carrascal *et al* (2003)	17.4	177	1996–1999	59 (14)	61	55	49	4
	Campos *et al* (2006)	17.4	368	2000–2003	61 (14)	63	61	45	19
India	Kattoor *et al* (2002)	3.8	215	1997–1999	60 (11)	75	49	41	No data
Iran	Abdirad *et al* (2007)	15.6	272	1969–2004	57 (11)	72	49	25	1
Japan	Koriyama *et al* (2004)	31.1	1927	1976–1995	62 (12)	64	50	43	37
Korea	Lee *et al* (2004)	41.4	621	1995–1996	55 (13)	67	49	53	32
Mexico	Herrera-Goepfert *et al* (2005)	7.9	330	1980–2000	58 (15)	52	48	63	4
Pakistan	Anwar *et al* (2005)	6.3	52	1996–2002	50 (15)	71	64	62	No data
Peru	Yoshiwara *et al* (2005)	21.2	256	1994–2001	65 (13)	49	51	50	14
All	—	—	5081	1949–2004	60 (13)	64	51	46	24

Abbreviations: ASR=age-standardised rate; GC=gastric cancer; s.d.=standard deviation; W=World.

a Excluding missing.

b National ASR as cases per 100 000 population (Ferlay *et al*, 2010).

c The diffuse subtype included the Japanese classifications por1, por2, sig, and LE, and the intestinal subtype included tub1, tub2, pap, and muc.

**Table 2:** Odds ratios of gastric cancer EBV positivity for age, sex, anatomic subsite, histologic type, and clinical stage of diagnosis

		**OR of EBV positivity (95% CI)**									
		**Age groups (years)**									
**Population**	**Overall EBV(+)**	**⩽35**	**36–45**	**46–55**	**56–65**	**>65 (reference)**	**Age effect per year**	**Sex (male)**	**Subsite (non- antrum)**	**Histologic type**^a^ **(diffuse)**	**Stage at diagnosis (advanced)**
*Brazil*											
Caucasians	10.3	2.1	2.0	1.3	3.7	1.0	1.0	3.0	5.1^b^	2.7	0.4
Blacks	16.0	1.0	0.4	1.5	0.03^c^	1.0	0.9	28.6^c^	0.1^c^	1.5	21.1^c^
Japanese Brazilians	4.7	7.8	3.3	1.6	0.3^c^	1.0	0.5^b^	0.4	1.0	2.3	5.8^c^
Chile	16.8	1.0	1.2	1.0	1.6	1.0	1.00	1.6	3.9^b^	4.4^b^	1.0
											
*China*											
Northern	6.1	0.2^c^	0.8	3.3	0.7	1.0	0.9	4.0	1.5	1.3	6.7^c^
Southern	9.1	6.9	2.9	2.0	1.3	1.0	0.7^b^	2.2	4.4^b^	12.2^b^	7.6^c^
											
*Colombia*											
Carrascal *et al*	13.0	1.8	2.9	2.4	0.9	1.0	0.7	3.5^b^	3.3^b^	1.2	15.8^c^
Campos *et al*	11.4	2.2	1.1	1.9	2.2	1.0	0.9	2.8^b^	1.6	2.5^b^	1.2
India	4.7	10.5	1.2	1.6	1.7	1.0	0.8	6.6^c^	6.1	3.6	No data
Iran	3.3	4.5	2.6	3.3	1.3	1.0	0.7	3.2	2.0	6.5^b^	3.3^c^
Japan	6.6	1.8	1.3	0.8	1.3	1.0	0.9	2.8^b^	3.6^b^	1.2	1.2
Korea	6.9	2.0	0.9	0.7	0.9	1.0	0.9	11.1^b^	3.4^b^	1.4	1.1
Mexico	7.3	0.1^c^	0.1^c^	0.6	0.8	1.0	1.8^b^	1.1	1.9	2.4	0.5
Pakistan	1.9	1.0^c^	10.0^c^	1.0^c^	1.0^c^	1.0	0.6	8.3^c^	1.0^c^	0.2^c^	No data
Peru	4.3	0.2^c^	1.4	2.7	0.3	1.0	0.9	1.3	2.9	1.8	0.7
Unadjusted Pooled OR		1.8 (1.1–2.8)	1.2 (0.8–1.8)	1.2 (0.8–1.6)	1.2 (0.9–1.6)	1.0	1.0 (0.98–1.00)	2.5 (2.0–3.3)	2.8 (2.2–3.6)	1.8 (1.5–2.3)	1.1 (0.9–1.5)
*I*^2^/*P*_Q_		0%/0.89	0%/0.89	0%/0.71	0%/0.86		17%/0.27	22%/0.21	0%/0.85	39%/0.1	0%/0.96

Abbreviations: CI=confidence interval; OR=odds ratio; EBV=Epstein-Barr virus.

a The diffuse subtype included the Japanese classifications por1, por2, sig, and LE, and the intestinal subtype included tub1, tub2, pap, and muc.

b *P*<0.05.

c 1% prevalence of EBV was assumed to allow calculation of population-specific ORs.

**Table 3:** Odds ratios (95% confidence intervals)^a^ of gastric cancer EBV positivity for males compared with females, by anatomic subsite and age

	**Anatomic subsite**	
	**Antrum**	**Non-antrum**
Age in years		
20	6.1 (2.1–17.9)	12.4 (4.6–33.4)
40	3.3 (1.6–6.6)	6.7 (3.7–12.1)
60	1.8 (1.1–2.9)	3.6 (2.4–5.3)
80	0.9 (0.5–1.8)	1.9 (1.0–3.6)

Abbreviation: EBV=Epstein-Barr virus.

a Estimates adjusted for geographic region, histologic type, diagnostic stage, and period of diagnosis.
